# Seroprevalence of IgG and IgE Antibodies Against *Anisakis* in the Presumably Healthy Population of the Canary Islands

**DOI:** 10.3390/antib14030060

**Published:** 2025-07-17

**Authors:** Eligia González-Rodríguez, Marta Rodero, J. Alberto Montoya-Alonso, Kevin M. Santana-Hernández, Myriam R. Ventura, Carmen Cuéllar, Eligia Rodríguez-Ponce

**Affiliations:** 1 Department of Animal Pathology and Production, Bromatology and Food Science and Technology, University of Las Palmas de Gran Canaria, 35017 Las Palmas de Gran Canaria, Spain; eligiagr55@gmail.com (E.G.-R.); alberto.montoya@ulpgc.es (J.A.M.-A.); kevin.santana106@alu.ulpgc.es (K.M.S.-H.); myriam.rodriguezventura@ulpgc.es (M.R.V.); eligia.rodriguezponce@ulpgc.es (E.R.-P.); 2 Department of Microbiology and Parasitology, Complutense University of Madrid, 28040 Madrid, Spain

**Keywords:** anisakiosis, seroprevalence, Canary Islands, IgG antibodies, IgE antibodies, ELISA, zoonoses, parasitic infection

## Abstract

Food-borne zoonoses, particularly anisakiosis caused by *Anisakis* spp., are an increasing public health concern due to the rising consumption of raw fish. Anisakiosis results from the ingestion of third-stage larvae of Anisakidae nematodes, with the genus *Anisakis* re-sponsible for approximately 97% of human cases. While regulatory protocols exist to minimize infection risk in commercial settings, domestic food preparation often lacks such safeguards, creating a gap in public health protection. In the Canary Islands, a major Spanish aquaculture region, farmed fish exhibit a low *Anisakis* prevalence, suggesting minimal risk from aquaculture products. In contrast, wild-caught fish demonstrate varia-ble parasitism, with recent studies reporting a 25% prevalence among commercial species. Methods: This study assessed *Anisakis* exposure in the Canary Islands by measuring specific IgG and IgE antibodies in 1043 serum samples collected from all seven islands between March 2014 and October 2015. ELISA assays detected anti-*Anisakis* antibodies, and the results were analyzed by age, sex, island, and isoclimatic zone. Results: Overall, 16.9% of samples were IgG-positive and 6.8% were IgE-positive. Seroprevalence was significantly higher in indi-viduals aged 60 years and above. Geographic heterogeneity was notable: La Palma had the highest IgG seroprevalence (35.3%), while El Hierro showed the highest IgE prevalence (16.3%). Temperate isoclimatic zones exhibited higher antibody prevalence than dry zones. These findings indicate variable *Anisakis* exposure across the Canary Islands, likely influenced by environmental and behavioral factors. Conclusions: The results highlight the need for targeted public health interventions to reduce the anisakiosis risk, particularly in regions and populations with elevated exposure.

## 1. Introduction

Zoonoses, especially food-borne diseases transmitted between vertebrate animals and humans, have become increasingly significant from a public health perspective. Globalization has facilitated the exchange of culinary practices and the introduction of diverse food products across the world; however, it has also introduced previously unrecognized risks associated with regional food sources [1]. In recent years, the consumption of raw fish has increased significantly as a dietary trend, accompanied by associated risks such as parasitic infections (e.g., anisakiosis) and allergic reactions.

Regulatory agencies, such as the U.S. Food and Drug Administration (FDA) and the European Food Safety Authority (EFSA), require specific preparation protocols (e.g., freezing) for raw fish dishes in commercial establishments to reduce the risk of infection. However, these safety measures are often lacking in domestic settings, where consumers preparing raw or undercooked seafood may be unaware of the need to use previously frozen fish unless it is thoroughly cooked. This discrepancy highlights a critical gap in food safety education and underscores the necessity for targeted public health initiatives to address safe seafood handling practices in the home.

Anisakidosis is a parasitic disease resulting from the ingestion of third-stage larvae (L3) of nematodes from the family Anisakidae, which are found in raw or undercooked fish and squid [2,3]. The genus *Anisakis* is responsible for the vast majority of human infections, accounting for approximately 97% of anisakidosis cases [3]. The presence of these larvae not only diminishes the commercial value of seafood products but also represents a significant public health risk if the parasites are not effectively eliminated during food processing [2].

Anisakiosis was first identified in humans in 1960 in the Netherlands and has since been documented globally [4,5]. In Europe, 236 cases were reported between 2000 and 2016, with the majority occurring in Spain and Italy. Spain currently has the highest number of reported anisakiosis cases in Europe and ranks second worldwide in diagnosed cases, following Japan [1,6,7].

*Anisakis* species have a cosmopolitan distribution and complex life cycle, involving eggs, four larval stages, and adults inhabiting marine mammal stomachs [8,9,10,11]. *Anisakis* eggs embryonate in seawater, releasing L2 larvae, which are ingested by crustaceans and develop into infective L3 larvae. L3 larvae migrate into fish or squid tissues, persisting in paratenic hosts without further development, and are transferred through predation [9,12,13,14]. Marine mammals ingest the infected hosts; L3 larvae mature to adults in their stomachs, completing the cycle [9].

Humans become accidental hosts for *Anisakis* by consuming raw or undercooked fish containing viable L3 larvae [3,4]. The clinical manifestations of anisakiosis depend on the site of larval invasion. Asymptomatic infection may occur when larvae remain within the gastrointestinal (GI) lumen without causing adverse effects. However, when larvae penetrate the GI mucosa, they can induce local irritation, severe epigastric pain, nausea, diarrhea, or low-grade fever. In some cases, more severe complications such as intestinal obstruction, perforation, peritonitis, and gastrointestinal bleeding may develop [5].

Larval invasion most commonly affects the stomach or intestines; however, on rare occasions, larvae may migrate to extra-GI sites, such as the throat or the peritoneal cavity. Gastric anisakiosis typically presents with symptoms within 1 to 12 h after ingestion, whereas symptoms of intestinal anisakiosis usually appear 5 to 7 days post-infection [5]. The nonspecific nature of these symptoms often leads to misdiagnosis, as they can resemble other GI conditions such as peptic ulcers, appendicitis, or inflammatory bowel disease.

Antigens released by live *Anisakis* larvae can elicit hypersensitivity reactions in humans. These allergic responses are mediated by IgE antibodies and may manifest clinically as urticaria, angioedema, asthma, or even anaphylaxis following the consumption of parasitized intermediate hosts [1,15].

The Canary Islands are the second largest region in Spain in terms of aquaculture fish production. In 2021, total aquaculture production in the Canary Islands was approximately 7000 tonnes, with fish farming, primarily of sea bass and sea bream, constituting the majority of this output. Specifically, in 2021, the Canary Islands accounted for 21% of Spain’s aquaculture sea bass production (4951 tonnes) and 8% of its sea bream production (720 tonnes).

A study conducted by the Spanish Aquaculture Business Association found no presence of *Anisakis* larvae in farmed fish, indicating that the risk of infection is negligible when consuming fish produced in controlled aquaculture environments. These findings support the conclusion that consumption of aquacultured fish significantly reduces the risk of *Anisakis* infection compared with wild-caught fish [16,17].

Analyses conducted over the past 15 years by the Parasitology Laboratory of the Faculty of Veterinary Medicine at ULPGC on fish from the Canary Islands coast (FAO Area 34), encompassing more than 20 commonly marketed ration-size species, have generally indicated low rates of *Anisakis* parasitism. However, a recent study examining 11 commercial fish species from the Canary Islands coast reported an overall prevalence of 25%, with only four of the studied species testing positive for *Anisakis* larvae [18].

The primary objective of this study was to provide an initial assessment of *Anisakis* infection in presumably healthy individuals in the Canary Islands. To this end, we analyzed the seroprevalence of specific IgG and IgE antibodies in serum samples collected from the population across the seven Canary Islands between March 2014 and October 2015. These samples, obtained from various diagnostic laboratories, had been utilized in a previous study [19]. This preliminary approach aimed to provide valuable information regarding the population’s sensitization to this parasite.

## 2. Materials and Methods

### 2.1. Subjects and Blood Samples

This study was conducted using a random sample of 1043 serum specimens collected from the population of the seven Canary Islands between March 2014 and October 2015. The samples were obtained from various diagnostic laboratories and had been utilized in a previous study [19]. All sera were stored at −80 °C until analysis. Where available, data regarding age, sex, and place of residence, specifically the municipality of origin, were recorded to assess potential isoclimatic influences.

The distribution of age and sex among the sampled individuals reflected the demographic structure of the Canary Islands’ population according to 2015 data [20]. Patients’ confidentiality and anonymity were strictly maintained throughout the study, and all participants provided written informed consent.

### 2.2. Anisakis spp. Antigens and Determination of Specific Antibodies

*Anisakis* antigens were prepared from L3 extracted from blue whiting (*Micromesistius poutassou*). The larvae were homogenized by sonication for 6 min (10 s pulses) and subsequently extracted in phosphate-buffered saline (PBS), as previously described by García-Palacios et al. [21].

Specific antibody levels were determined by an enzyme-linked immunosorbent assay (ELISA). Microtiter plates (Costar, Corning, NY, USA) were coated with 10 µg/mL of *Anisakis* larval antigen on the first four rows, while the remaining rows were left uncoated to serve as controls for nonspecific binding. Plates were incubated overnight at 4 °C. The following day, the plates were washed three times with 0.05% PBS-Tween 20 and blocked with 200 µL per well of 0.1% bovine serum albumin (BSA) in PBS for 1 h at 37 °C. After additional washing, 100 µL of the serum samples was added in duplicate, diluted 1:100 for IgG and 1:2 for IgE in PBS-Tween with 0.1% BSA, and incubated at 37 °C for 2 h. For IgG detection, plates were washed and incubated with 100 µL per well of goat anti-human IgG peroxidase-conjugated antibody (Invitrogen, Thermo Fisher Scientific, Waltham, MA, USA), diluted 1:8000 in PBS-Tween with 0.1% BSA, for 1 h at 37 °C. For IgE detection, 100 µL per well of a human IgE-specific monoclonal antibody (Ingenasa, Gold Standard Diagnostics, Madrid, Spain), diluted 1:1000, was added and incubated as above. After washing, 100 µL per well of cross-adsorbed goat anti-mouse IgG1 HRP (Invitrogen, Thermo Fisher Scientific, Waltham, MA, USA), at the same dilution, was added for 1 h at 37 °C. For both protocols, after a final wash, 100 µL per well of the substrate (o-phenylenediamine, Sigma, St. Louis, MO, USA) at 0.04% in a phosphate–citrate buffer (pH 5.0) with 0.04% hydrogen peroxide was added. The reaction was stopped with 50 µL of 3N H_2_SO_4_, and absorbance was read at 490 nm using a HEALES MB-580 spectrophotometer [22,23].

### 2.3. Sample Classification

Information collected for each serum sample included age, gender, and isoclimatic zone. Samples were categorized into five age groups, each spanning a 15-year range. The final group comprised individuals aged 60 years and older.

### 2.4. Climate Classification

Samples were classified according to the climatological zones corresponding to the municipality of origin of each individual in the archipelago, using the Köppen classification system, also known as the Köppen–Geiger classification, as described by Rodríguez-Ponce et al. [24]. This system categorizes climate types according to the average monthly precipitation and temperature, with temperature and precipitation thresholds established primarily for their influence on vegetation distribution and human activity.

In the Canary Islands, the Köppen classification identifies several climate types. These include dry zones, subdivided into hot desert (BWh) and hot semi-arid (BSh) subclimates, and temperate zones (mesothermal), which have higher precipitation, mainly during the coldest months, and average winter temperatures below 18 °C. The temperate zones are further subclassified into warm-summer Mediterranean (Csb) and hot-summer Mediterranean (Csa) subclimates. Islands such as El Hierro, La Gomera, and La Palma are where these climatic varieties are most frequently observed [25].

### 2.5. Statistical Analysis

The ELISA assay produced four readings per sample: two wells sensitized with the *Anisakis* antigen (A1 and A2) and two wells not sensitized (B1 and B2, containing only BSA). The final result (FR) for each sample was calculated using the following formula:

FR = ((A1 + A2) − (B1 + B2))/2

Samples were considered positive if their FR was equal to or greater than the sum of the mean plus one standard deviation of all samples. Given our focus on a presumably healthy population, this threshold optimally balances sensitivity (detecting low-level exposures) and specificity (reducing false positives from cross-reactivity).

For statistical analysis, Student’s *t*-test was used to compare quantitative variables when normality was confirmed by the Kolmogorov–Smirnov test. If normality was not met, the Mann–Whitney *U* test was applied. Statistical significance was defined as *p* < 0.05. Data analysis was performed using RStudio^®^ (version 2022.02.1 Build 461, R Foundation for Statistical Computing, Vienna, Austria), Microsoft Excel^®^ Office 365^®^ (Microsoft Corporation, Redmond, WA, USA) and GraphPad Prism version 6.0 (GraphPad Software, San Diego, CA, USA).

## 3. Results

Table 1 presents the distribution of samples by age group for each island.

The results indicated that 176 out of 1043 samples (16.9%) tested positive for IgG antibodies, while 70 out of 1036 samples (6.8%) were positive for IgE antibodies. For comparative analysis, the samples were categorized into five age groups.

**Figure 1:**
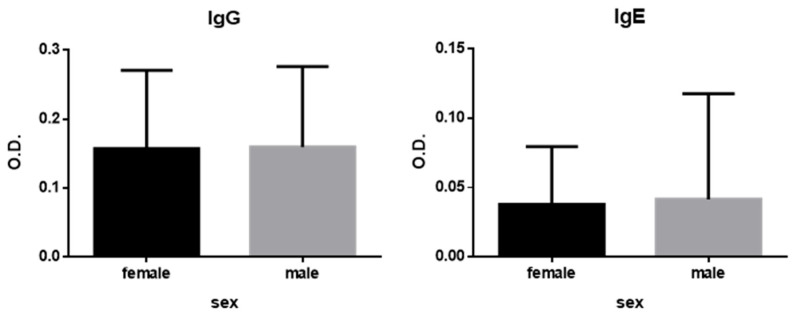
Mean and standard deviation of IgG and IgE anti-*Anisakis* antibody levels by sex. Group comparisons were performed using Student’s *t*-test for normally distributed data and the Mann–Whitney *U* test for non-normally distributed data.

Figure 1 presents the results stratified by sex. Based on statistical analysis of the data, no significant differences were observed between male and female participants within the studied population.

**Figure 2:**
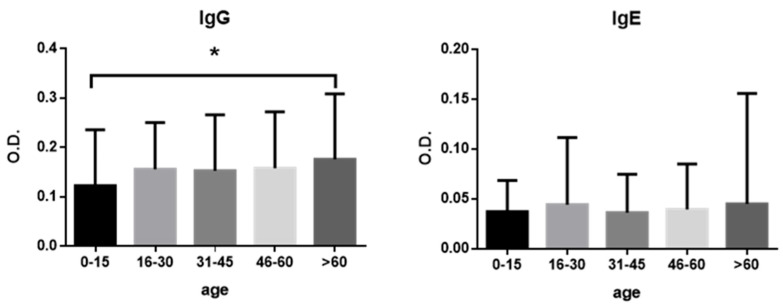
Mean and standard deviation of IgG and IgE anti-*Anisakis* antibody levels by age group. Group comparisons were performed using Student’s *t*-test for normally distributed data and the Mann–Whitney *U* test for non-normally distributed data. * *p* < 0.05.

However, the proportion of seropositive individuals in the 60+ age group was significantly higher (*p* < 0.05) compared with the 0–15 year age group (Figure 2). The mean age of seropositive subjects was 47.5 years.

**Figure 3:**
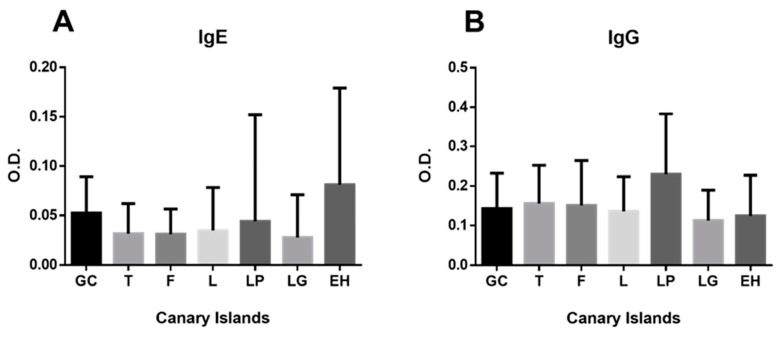
Mean and standard deviation of IgG (**B**) and IgE (**A**) anti-*Anisakis* antibody levels by island (GC: Gran Canaria; T: Tenerife; F: Fuerteventura; L: Lanzarote; LP: La Palma; LG: La Gomera; EH: El Hierro). Group comparisons were performed using Student’s *t*-test for normally distributed data and the Mann–Whitney *U* test for non-normally distributed data. Significant differences are described in the text.

Significant variations in seroprevalence percentages were observed across the Canary Islands for different antibody isotypes (Figure 3).

For IgG anti-*Anisakis* antibodies, La Palma exhibited the highest seroprevalence (35.3% positive sera, *p* < 0.0001), followed by Fuerteventura (18.7%), Tenerife (12%), Lanzarote (11.8%), Gran Canaria (11.7%), El Hierro (8.8%), and La Gomera (6.3%) (Table A1).

IgE anti-*Anisakis* distribution showed distinct patterns: El Hierro had the highest prevalence (16.3% positive sera); Gran Canaria followed with 13.8%. Lower rates occurred in Lanzarote (6.5%), La Palma (4.9%), Tenerife (4.1%), La Gomera (3.1%), and Fuerteventura (1.9%) (Table A1). Statistical comparisons revealed that El Hierro’s IgE levels differed significantly from those of Lanzarote and La Gomera (*p* < 0.0001), Tenerife and Fuerteventura (*p* < 0.001), and La Palma (*p* < 0.05). Gran Canaria showed significant IgE differences versus Tenerife, Fuerteventura, Lanzarote, La Gomera (*p* < 0.0001), and La Palma (*p* < 0.05).

**Figure 4:**
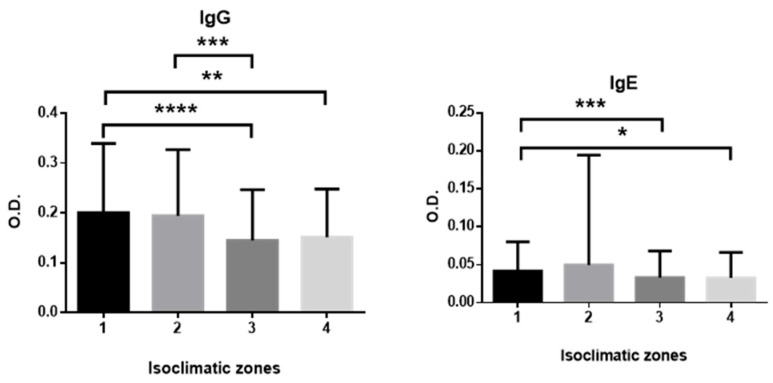
Mean and standard deviation of IgG and IgE anti-*Anisakis* antibody levels by isoclimatic zone: 1, mild temperate (Tm); 2, cold temperate (Tc); 3, dry desert (Dd); 4, dry steppe (Ds). Group comparisons were performed using Student’s *t*-test for normally distributed data and the Mann–Whitney *U* test for non-normally distributed data. * *p* < 0.05; ** *p* < 0.01; *** *p* < 0.001; **** *p* < 0.0001.

Figure 4 summarizes the differences in prevalence according to the isoclimate of origin of the samples. For both IgG and IgE anti-*Anisakis* antibodies, the highest prevalence was observed in temperate (T) climates. In the Tm isoclimate, 218 samples were analyzed, with 61 testing positive for IgG anti-*Anisakis* (28%), while in the Tc isoclimate, 20 out of 94 samples were positive (21.3%). In contrast, dry climates (D) showed the lowest prevalence: the Dd isoclimate had 69 positives out of 437 samples (15.8%), and the Ds isoclimate had 15 seropositives out of 150 samples (10%) (Table A1). The temperate climates correspond to inland areas of the islands at higher altitudes, whereas dry climates are prevalent in coastal regions and islands lacking significant elevation.

For IgE anti-*Anisakis* antibodies, 214, 93, 435, and 150 samples were analyzed for the Tm, Tc, Dd, and Ds isoclimatic zones, respectively, with seropositivity rates of 7%, 6.5%, 4.8%, and 3.3% (Table A1). Significant differences in IgG and IgE antibody levels among isoclimatic zones are shown in Figure 4.

## 4. Discussion

In 2022, following the COVID-19 pandemic, the Canary Islands received 14,617,383 international tourists [26]. This high influx of visitors, together with the archipelago’s strategic geographical location, contributes to the Canary Islands’ status as a region with considerable potential for the transmission of various human parasites [19,27]. In 2017, it was estimated that Spain experienced approximately 8000 cases of anisakiosis annually; however, the true incidence is likely higher due to underdiagnosis and misdiagnosis resulting from the nonspecific clinical presentation and the occurrence of asymptomatic cases. Notably, in 2013, the Canary Islands reported the lowest incidence of anisakiosis among Spain’s autonomous communities, with one case per 100,000 inhabitants [28]. This low incidence stands in contrast to the higher seroprevalence rates observed in the present study.

Humans are accidental hosts of *Anisakis*, typically becoming infected through the ingestion of raw or undercooked fish. Both the act and frequency of consuming such products are confirmed as the primary risk factors for anisakiosis [29,30,31]. In Spain, the consumption of anchovies in vinegar (“*Boquerones en vinagre*”) is particularly associated with cases of anisakiosis, as this preparation involves marinated but not fully cooked fish. Moreover, failures in freezing procedures have contributed to outbreaks [31,32,33]. For example, in Madrid, a region with a high incidence of anisakiosis, “*Boquerones en vinagre*” constitute a significant part of the diet, and the practice of consuming lightly cooked or marinated fish is more prevalent, thereby increasing the risk of infection [28,31]. In contrast, Galicia, despite its high overall consumption of fish and seafood, exhibits a very low seroprevalence, likely attributable to a preference for thoroughly cooked fish dishes [31,34].

In the Canary Islands, per capita fish consumption is the lowest in Spain [35]; nevertheless, anchovies remain an important component of the local diet and are consumed in various forms, including fresh, canned, semi-preserved, and pickled [36]. This diversity in preparation methods, particularly the consumption of “*Boquerones en vinagre*” or other lightly processed forms, constitutes a significant risk factor for *Anisakis* exposure in the region. However, traditional Canarian cuisine also includes dehydrated salted fish and canned seafood, which are less likely to transmit *Anisakis* due to preservation processes that eliminate the parasite. These culinary practices may help explain why the seroprevalence observed in this study is lower than that reported in other regions of Spain, despite the presence of certain high-risk foods in the local diet.

Seroprevalence studies conducted in various countries and regions across different continents demonstrate considerable variability. IgE positivity is unequivocally linked to allergic sensitization, reflecting an active immunological response with potential clinical manifestations. IgG positivity, however, indicates prior antigen exposure without implying current allergic disease. Its presence may signify immunotolerance, subclinical exposure, or resolved infection, but not necessarily pathology. For example, a study conducted in Cali, Colombia, where hygienic conditions were suboptimal, reported a very low seroprevalence of 1.3% [37]. In contrast, research conducted in Niterói, Brazil, found a seropositivity rate of 20.9% for IgE antibodies, suggesting that IgG seroprevalence could be even higher [38]. These differences may be influenced by factors such as dietary habits, local awareness, and diagnostic practices regarding anisakiosis, as well as the specific antibody isotype measured in each study.

A study conducted in South Korea reported a seroprevalence of 5.0% for anti-*Anisakis* IgE antibodies using ELISA, a value very similar to that observed in the present study [39]. In Europe, seroprevalence rates also vary considerably, with reported values ranging from 2% in Croatia [29,40] to 0.4% in Norway, despite Norway’s markedly different climate and dietary patterns, as well as its very high per capita fish consumption [41,42].

Spain and Italy, both considered endemic countries for anisakiosis, exhibit similar seroprevalence rates, with estimates around 7% [6], which closely align with the data observed in the present study. In Spain, seroprevalence varies markedly by region and isotype measured, with limited data available for IgG. For example, Galicia has reported a low seroprevalence of 0.43% [34], while Madrid, which historically has the highest mean rate of anisakidosis hospitalizations in the country (9.17 hospitalizations per million inhabitants) [7], showed an IgE seroprevalence of 12.4% in earlier studies (2001–2002) [43], approximately double the rate found in the present study. Over the past 20 years, the prevalence of anisakiosis in Madrid has decreased dramatically. By 2021–2023, this prevalence had declined to 2.2%, representing a reduction of more than 80% [31]. This significant decline is attributed to the implementation of preventive legislation in Spain, notably European Regulation (EC) No. 853/2004 and Royal Decree 1420/2006, which mandate measures such as the freezing of fish intended for raw or undercooked consumption. Despite the continued consumption of raw fish, these control measures, along with increased public awareness, have proven effective in reducing infection rates. The current prevalence in Madrid is now comparable with, or even lower than, that observed in other European countries.

Even higher rates have been documented in Antequera, a city in the coastal province of Malaga, where IgE seroprevalence was estimated at 22.4% [44]. These findings highlight significant geographic variability in anisakiosis-related seroprevalence within Spain, influenced by regional dietary habits and exposure risks.

Morocco, which is geographically close to the Canary Islands and shares the same marine waters, showed a seroprevalence of 5.1% for anti-*Anisakis* IgE antibodies in a study conducted in 2012 in the northern provinces of Tetouan and Tangier [45]. This finding highlights the similarity in sensitization rates between Morocco and the Canary Islands.

When analyzing the overall results for the Canary Archipelago, La Palma stands out with the highest prevalence of anti-*Anisakis* specific IgG at 35.3%, compared with 16.9% for the archipelago as a whole. This elevated prevalence cannot be attributed to seafood consumption, as a study on mercury intake conducted in 2008 [46] identified La Palma as the island with the lowest fish consumption. The second highest prevalence was observed on Fuerteventura (18.7%), followed by Tenerife (12%), which, in contrast, leads the archipelago in fish consumption. This geographic heterogeneity in immune response patterns suggests the presence of potential environmental or behavioral factors influencing *Anisakis* exposure and sensitization across the archipelago.

In the results of this study, no significant differences were observed according to sex. This finding is consistent with previous studies conducted in Croatia, Morocco [45], and Italy [47], where no differences were reported between males and females in the populations studied. Conversely, a retrospective study from France [48] identified a significant female predominance, a trend also observed in South Korea [39]; however, these studies focused on clinical cases of anisakiosis rather than immunological sensitization in the general population.

This study indicates a likely correlation between age and *Anisakis* prevalence, as IgG seroprevalence increased with age in the population analyzed, although IgG seropositivity alone does not denote clinical disease [31]. Similar trends have been reported in Italy, where most cases occurred in individuals in their thirties and forties [47]. In Morocco, the highest sensitization was observed in the 31–43 year age group, but a decrease in sensitized individuals was noted among those older than 57 years [45]. In Spain, the majority of hospitalizations for anisakiosis between 1997 and 2015 were recorded in individuals aged 45 to 64 years [7]. In contrast, IgE seroprevalence did not show a significant association with age in this study [31].

Climatic factors play a critical role in the epidemiology of *Anisakis* infection, influencing both the prevalence and distribution of the parasite in marine environments. Temperature and other environmental variables affect larval mobility, survival, and transmission dynamics, with higher temperatures facilitating larval movement and potentially increasing infection rates in fish hosts [2,9,10,13,49]. Seasonal and regional differences in climate can also impact the abundance and distribution of intermediate and definitive hosts, as well as the prevalence of *Anisakis* in fish populations consumed by humans. Consequently, including climate as a variable in seroprevalence studies of anti-*Anisakis* antibodies is essential for accurately assessing the exposure risk and understanding spatial variations in infection within human populations, such as those in the Canary Islands.

The notably high seroprevalence observed on the island of La Palma, where areas with dry climates are very limited, is particularly striking. In contrast, Fuerteventura, the island with the second highest prevalence, consists exclusively of dry climate zones. These findings suggest that the influence of isoclimatic zones on *Anisakis* seroprevalence is complex and not straightforward, underscoring the need for further studies to elucidate the relationship between climate and the prevalence of *Anisakis* in the Canary Islands.

Epidemiological data on *Anisakis* species in fish from the waters of the Canary Archipelago are limited. A previous study analyzing 475 fish representing 33 different commercial species from markets on the island of Gran Canaria, most of which originated from FAO Area 34 (87.4%) [27], reported an overall prevalence of 5.5% for larvae of the family Anisakidae. An additional 3.6% of the fish originated from FAO Area 27, where the presence of *Anisakis* is typically more pronounced. Among the fish examined, 82.1% belonged to the families Sparidae (61%), Scaridae (10%), Clupeidae (6.5%), and Serranidae (4.6%), while the remaining 17.9% comprised species from the families Scombridae, Sphyraenidae, Haemulidae, Carangidae, Mullidae, Balistidae, and Triglidae.

Martín-Carrillo et al. [18] conducted a study on fish species of economic importance, collected both from markets and directly from the Canary coast, and reported an overall *Anisakis* spp. prevalence of 25%, a result notably higher than that observed in the previous study focused solely on the island of Gran Canaria. Molecular analyses identified five of the ten recognized *Anisakis* species, with *A. simplex* (*s.s*.) and *A. pegreffii* being the species most frequently implicated in human anisakiosis cases. The fish species testing positive for *Anisakis* primarily belonged to the Scombridae and Merlucciidae families, which likely accounts for the differences in prevalence observed between the two studies, as these families are known to exhibit higher infection rates [18,49].

Accurate species identification is further complicated by the mislabeling of fish products, particularly given that *Scomber scombrus* has not been recorded in the waters off the Canary Islands, thereby increasing the risk of confusion when labeling is incorrect. In light of these challenges, it is essential to continue the molecular identification of anisakid species present in the main fish species commonly consumed. This approach will enable more precise monitoring of the health status of fishery products and facilitate the assessment of the public health impact associated with the presence of anisakids [18,50].

The principal limitation of this study lies in the absence of critical epidemiological information, particularly detailed dietary histories, clinical status, and occupational exposure data. The retrospective analysis of archived serum samples, which were not originally collected for the purpose of *Anisakis* investigation, introduces selection bias and restricts the generalizability of the findings. Furthermore, the lack of information on key risk factors significantly impedes the identification of associations between exposure and sensitization. The cross-sectional study design further limits the ability to draw causal inferences, underscoring the need for longitudinal data to accurately assess trends in seroprevalence. Additionally, small sample sizes within certain geographic and climatic subgroups reduce the statistical power, thereby limiting the robustness of comparative analyses. To address these shortcomings, future research should employ prospective study designs with purpose-collected samples and comprehensive data collection, including detailed dietary questionnaires, clinical histories, and occupational exposure assessments. Increasing sample sizes, particularly in underrepresented regions, and conducting longitudinal follow-up would enhance the validity, geographic resolution, and clinical relevance of *Anisakis* research in the Canary Islands population.

## 5. Conclusions

This study represents the first large-scale seroepidemiological assessment of *Anisakis* exposure in the presumably healthy population of the Canary Islands. The results reveal a notable seroprevalence of anti-*Anisakis* IgG (16.9%) and IgE (6.8%) antibodies, indicating substantial exposure and sensitization to *Anisakis* spp. among inhabitants of the archipelago. The data demonstrate pronounced geographic heterogeneity, with the highest IgG seroprevalence observed in La Palma and the highest IgE prevalence in El Hierro, suggesting that local environmental or behavioral factors may influence the risk of exposure and allergic sensitization.

Furthermore, the study identifies a significant increase in seroprevalence with age, particularly among individuals over 60 years, which may reflect cumulative lifelong exposure to *Anisakis* antigens through dietary habits. No significant differences were detected between sexes, indicating that exposure risk is likely related to share cultural or dietary practices rather than gender-specific behaviors.

Analysis by isoclimatic zone revealed higher seroprevalence rates in temperate regions compared with dry zones, supporting the hypothesis that environmental conditions may modulate the risk of *Anisakis* infection and sensitization, possibly through their influence on local fish species composition and consumption patterns.

These findings underscore the importance of public health education regarding the risks associated with the consumption of raw or undercooked fish, particularly in regions with higher prevalence rates. The results also highlight the need for further research to elucidate the specific factors contributing to geographic and climatic differences in *Anisakis* exposure. Finally, the study advocates for the implementation of targeted preventive strategies, including consumer awareness campaigns and continuous monitoring of *Anisakis* prevalence in both wild and farmed fish, to reduce the burden of anisakiosis and related allergic disorders in the Canary Islands population.

As future work, we plan to conduct prospective studies incorporating clinical assessment, detailed dietary surveys, and longitudinal follow-up of seropositive individuals to better elucidate exposure pathways and clinical outcomes. Given the public health implications of *Anisakis* infection, we also recommend consideration of targeted educational campaigns, enhanced food labeling, and regulatory measures such as mandatory freezing of high-risk fish products to reduce consumer risk in the Canary Islands.

## Figures and Tables

**Table 1:** Distribution of samples by island, antibody type, and age in the Canary Islands.

Island	N (IgG)	N (IgE)	Mean Age ± SE (Years)	Min Age	Max Age
Gran Canaria (GC)	94	94	40 ± 2	4	75
Tenerife (TF)	217	217	49 ± 1	2	90
Fuerteventura (FV)	214	214	42 ± 1	2	73
Lanzarote (LZ)	187	185	49 ± 1	13	81
La Palma (LP)	187	182	47 ± 1	8	80
La Gomera (G)	64	64	43 ± 2	10	83
El Hierro (H)	80	80	44 ± 2	2	82
Total	1043	1036	45.5 ± 0.5	2	90

N: number of samples analyzed for each antibody; SE: standard error.

## Data Availability

The original contributions presented in this study are included in the article. Further inquiries can be directed to the corresponding authors.

## Appendix A

**Table A1:** Prevalence of anti-*Anisakis* IgG and IgE antibodies in analyzed samples by island and isoclimatic zone in the Canary Islands.

	% IgG Positive	% IgE Positive
Gran Canaria (GC)	11.7	13.8
Tenerife (TF)	12	4.1
Fuerteventura (FV)	18.7	1.9
Lanzarote (LZ)	11.8	6.5
La Palma (LP)	35.3	4.9
La Gomera (G)	6.3	3.1
El Hierro (H)	8.8	16.3
Temperate, mild (Tm)	28	7
Temperate, cold (Tc)	21.3	6.5
Dry desert (Dd)	15.8	4.8
Dry steppe (Ds)	10	3.3
